# Integrative oncology for breast cancer patients: introduction of an expert-based model

**DOI:** 10.1186/1471-2407-12-539

**Published:** 2012-11-21

**Authors:** Gustav J Dobos, Petra Voiss, Ilka Schwidde, Kyung-Eun Choi, Anna Paul, Barbara Kirschbaum, Felix J Saha, Sherko Kuemmel

**Affiliations:** 1 Department of Internal and Integrative Medicine, Kliniken Essen-Mitte, academic teaching hospital of the University of Duisburg-Essen, Am Deimelsberg 34 a, 45276 Essen, Germany; 2 Department of Senology/ Breast Center, Kliniken Essen-Mitte, Evang. Huyssens Stiftung/Knappschaft GmbH, Henricistr. 92, 45136, Essen, Germany; 3 Chair of Complementary and Integrative Medicine, Alfried Krupp von Bohlen und Halbach-Foundation, University of Duisburg-Essen, Kliniken Essen-Mitte, Knappschafts-Krankenhaus, Am Deimelsberg 34 a, 45276 Essen, Germany

**Keywords:** Integrative oncology, Integrative medicine, Complementary and alternative medicine, Holistic care

## Abstract

**Background:**

Malignant breast neoplasms are among the most frequent forms of cancer in the Western world. Conventional treatment of breast cancer may include surgery, hormonal therapy, chemotherapy, radiation and/or immunotherapy, all of which are often accompanied by severe side effects. Complementary and alternative medicine (CAM) treatments have been shown to be effective in alleviating those symptoms. Furthermore, with patient survival rates increasing, oncologists, psychologists and other therapists have to become more sensitive to the needs of cancer survivors that go beyond than the mere alleviation of symptoms. Many CAM methods are geared to treat the patient in a holistic manner and thus are also concerned with the patient’s psychological and spiritual needs.

**Discussion:**

The use of certain CAM methods may become problematic when, as frequently occurs, patients use them indiscriminately and without informing their oncologists. Herbal medicines and dietary supplements, especially, may interfere with primary cancer treatments or have other detrimental effects. Thus, expertise in this highly specialized field of integrative medicine should be available to patients so that they can be advised about the benefits and negative effects of such preparations and practices.

Being a beneficial combination of conventional and CAM care, integrative oncology makes possible the holistic approach to cancer care. The concept of integrative oncology for breast cancer is jointly practiced by the Department of Internal and Integrative Medicine, Kliniken Essen-Mitte, academic teaching hospital of the University of Duisburg-Essen, and the Breast Center at Kliniken Essen-Mitte in Germany. This model is introduced here; its scope is reviewed, and its possible implications for the practice of integrative medicine are discussed.

**Summary:**

Evidence-based integrative care is crucial to the field of oncology in establishing state-of-the-art care for breast cancer patients.

## Background

In 2008 alone, about half of a million women worldwide died from breast cancer, one of the most frequent forms of invasive cancer in women (International Agency for Research on cancer, 2008). In recent years, state-of-the-art breast cancer care has increasingly included the emerging concept of integrative medicine (IM) (in Great Britain “integrated medicine”).

### The concept of integrative oncology (IO)

**Figure 1:**
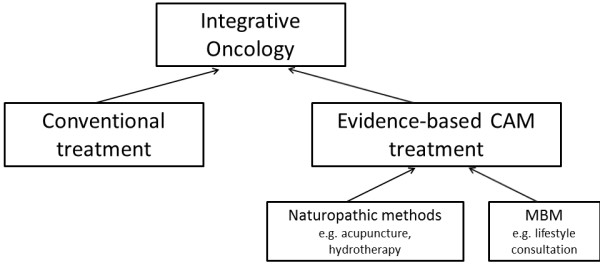
Components of Integrative Oncology.

The definition of IM in general and integrative oncology (IO) in particular, as well as the use of their treatment modalities, depends on the country and/or culture within which they are practiced [1,2]. Both IM and IO combine conventional with other medical approaches that have been shown to be safe and effective [3] (see Figure 1). Phrases such as “holistic”, “complementary”, or “alternative” refer to the methods advocated by “other medical approaches”, which include the multitude of diverse medical and health care systems, practices, and products not generally considered part of conventional medicine. The *Arbeitsgemeinschaft Gynäkologische Onkologie e.v.* (AGO), which establishes yearly guidelines for the treatment of breast cancer in Germany, defines the terms “complementary” and “alternative” as follows: “CAM, […] comprises both alternative therapies that are used instead of conventional, optimally scientifically based medicine, and complementary methods that are used in addition to conventional methods with proven efficacy. Conventional clinicians tend to approve of a complementary approach more readily than one of the other options. However, if complementary approaches are administered simultaneously with conventional therapies, there is always the risk that they will interfere with the standard treatment, e.g., in the form of drug interactions with partially incalculable outcomes.” Integrative oncology combines the best practices of conventional and complementary oncological therapy, uniting them into one, holistic concept. With the awareness that the two therapeutic methods may occasionally interfere with each other, the best solution is aimed-at.

The Concerted Action for Complementary and Alternative Medicine Assessment in the Cancer Field (CAM-Cancer) project divides the CAM approaches into the following categories ( http://www.cam-cancer.org):

• alternative medical systems (e.g., homeopathy, traditional Chinese medicine),

• biologically based practices (e.g., herbs, vitamins, food),

• energy medicine (e.g., Reiki),

• mind-body medicine, (e.g., meditation, autogenic therapy, progressive muscle relaxation), and

• manipulative and body-based practices (e.g., massage).

In 1998, the National Center for Complementary and Alternative Medicine (NCCAM) was established as part of the National Institutes of Health (NIH) in the United States. Allotted over 120 million dollars for CAM and Mind-body medicine (MBM) research in 2008 alone, it is the world's leading research facility for CAM ( http://www.nccam.nih.gov). CAM methods are now widespread and are even included in the curricula of Western universities and institutions [4], including such prestigious ones as Stanford University Medical School, the Harvard Medical School, and the Memorial Sloan-Kettering Cancer Center in New York, which has an Integrative Medicine Service [5].

### The field of application of IO

An important feature of IM is that it actively involves patients in their own treatment, thus encouraging them to take responsibility for maintaining their own health. There are at least two outstanding reasons for integrating CAM into cancer care. First, patients with cancer often experience multiple symptoms not only from the cancer but also from its treatment [6]. Conventional cancer therapies such as surgery, chemotherapy, immunotherapy and/or radiation are generally life-prolonging, but they may also be accompanied by serious side effects such as pain, nausea, fatigue, sleep disturbances, oral and/or gastrointestinal ulceration and inflammation, and manifest psychological disorders [3]. As a rule these side effects are concomitant with the primary cancer therapy, beginning with the first treatments, and are not satisfactorily alleviated by conventional means. Although they may vary in type and severity over time, they definitely restrict the physical, psychological, and social functions of the affected patients.

Preliminary findings indicate that supportive CAM methods may be of value from the time a patient receives the diagnosis of cancer on to the time of their rehabilitation and recovery [5]. Instead of expecting to cure their disease, patients learn to deal with it, using CAM to strengthen their immune system, relieve pain, and manage the side effects from the disease or its treatment, often working with a supportive practitioner [7]. In particular, the fatigue and nausea caused by primary cancer treatment may be alleviated, and recovery from chemotherapy may be hastened [8-10]. In addition, acupuncture, mind-body techniques, and massage may help relieve side effects and improve patients’ physical and emotional well-being. At present, about one in three breast cancer patients discontinues her hormone treatments against medical advice; in such cases CAM interventions can improve compliance by reducing hot flashes or joint pain [11-13].

A special advantage of CAM interventions is that they are particularly well suited to treat patients in a holistic manner, including dealing with their psychological and spiritual needs [14]. Holistic approaches, which are often individualized, focus on patient orientation, the patient–doctor or –therapist relationship, and on understanding the patient’s perspective by means of multimodal concepts. Since patient survival rates have increased, oncologists, psychologists, and other therapists have to become more sensitive to patient needs that go beyond the mere alleviation of symptoms.

Not surprisingly, about 40% of cancer survivors use a CAM method, and 18% use multiple CAM therapies (the National Health Interview Survey, NHIS, 2002 and 2007). Among women with breast cancer, the rate of CAM use is even up to 75% [15]. The most frequently used CAM therapies among cancer survivors are herbal and other natural products (the National Health Interview Survey, NHIS, 2002 and 2007). Patients are likely to be motivated to use CAM treatments by the benefits they perceive to result from CAM, a desire to feel more in control of their health, or a strong belief in CAM ( http://www.cam-cancer.org).

Unfortunately, the majority of patients use CAM methods indiscriminately and without informing their oncologists [1], exposing themselves to possible detrimental effects. These are due especially to a CAM treatment interacting with their chemotherapy or endocrine treatment. Medical professionals repeatedly criticize that many CAM methods are poorly supported by scientific evidence [16]. Therefore, expertise in this field of IM is needed so that patients can be advised about the benefits and negative effects of such CAM methods and preparations. This article introduces a German expert-based model for IO that combines mainstream medicine and CAM for the care of breast cancer patients.

### IM in Germany

In 2004, the first German Chair for Complementary and Integrative Medicine was established at the University of Duisburg-Essen (chair held by GJD). Unlike in the United States, research funding for CAM research in Europe is very limited. In addition the clinical practice of CAM in Europe is based more on patient request. In the meantime, a number of German university departments are focusing on CAM research thanks to third-party funds. In a cross-sectional study of medical schools in German-speaking countries, the majority (40%) of decision makers had a positive opinion of CAM and were in favor of integrating CAM into medical school curricula, especially in research and education [17]. However, only a small percentage of institutions and hospitals have actually integrated CAM methods into their programs. This is not in line with the prevalence of usage of CAM in the population.

The use of CAM in Western countries has increased steadily over the past decades. Whereas several surveys on the use of different CAM therapies and on predictors of CAM usage have been carried out in the USA, little is known about the use of CAM in Europe [1]. According to a European survey on CAM use in cancer patients, the most commonly used CAM therapies were herbal medicines and remedies, homeopathy, vitamins/minerals, medicinal teas, spiritual therapies, and relaxation techniques. Predictors for CAM use are a young age, female sex, and a higher level of education [1]. The most frequently used methods in children with cancer were homeopathy, dietary supplements, and anthroposophic medicine (AM), including mistletoe therapy [18].

However, the data on CAM use in the care of German cancer patients is still insufficient, there being more specific data on CAM use in general. In the year 2000, the Allensbach Institute for Public Opinion Polling estimated from 2111 face-to-face interviews that 50% of the German population had personally used at least one CAM method at some time [19]. Two years later, 73% of the German population used CAM [19]. Popular methods included naturopathy (48%), autogenic training (29%), and meditation (21%), as well as acupuncture (25%) and acupressure (12%). The rates are highly dependent on the patient’s symptoms: Patients presenting with headache most frequently used acupuncture (58.3%), massage (46.1%), and relaxation techniques (42.4%) [20]. General medicine practices most commonly employ neural therapy, phytotherapy, and acupuncture (ca. 65%, 53%, and 38%) [21]. Older German adults most frequently used acupuncture/traditional Chinese medicine (21%), homeopathy (21%), movement therapies/physical exercises (19%), osteopathy/chiropractic (12%), herbs/phytotherapy (7%), diets/specific food recommendations (6%), and foot reflexology (5%) [22]. According to a cross-sectional survey on consecutive patients visiting the Comprehensive Cancer Center in Munich, Germany, the most common CAM treatments were diet (40%), physical exercise (28%), and dietary supplements (28%) [23]. While 52% used at least one CAM method, only 34% described themselves as well informed about CAM. Interestingly, there are indications that involvement in self-support groups and leisure activities are associated with CAM use in breast cancer patients [24].

It should be noted that the differences in percentages may not be as specific as they might seem at first glance. In a survey of the Robert-Koch Institute on CAM use in Germany the authors remarked that terms such as alternative, complementary, or naturopathic medicine are highly unspecific, and divergent survey outcomes may be the result of differing individual views on what these terms exactly include [25].

Nevertheless, the relevance of specific CAM interventions may vary among different countries, depending on the socio-cultural background, availability of approaches, financial resources, and specific individual preferences. German-speaking countries have the highest prevalence of CAM use among the European countries [26]. This may be explained by the fact that homeopathy, AM, and naturopathy have their origins in the German-speaking countries. Thus, many home remedies and self-medication practices are rooted in the health care approaches of these countries [26].

AM was introduced by Steiner and Wegman in the early 20th century. Homeopathic remedies, whole plant extracts, therapeutic eurythmy, art therapies, rhythmical massage, and biographical counseling are examples of AM methods. The AM treatment with the highest relevance for cancer patients may be the use of mistletoe. Homeopathy is an alternative medical system from Germany, introduced by the German physician Samuel F.S. Hahnemann. It is based on the idea that a substance that causes symptoms of a certain disease in healthy individuals can be used to treat patients with that disease. Kneipp medicine, conceived by Sebastian Kneipp, consists of naturopathic treatments such as hydrotherapy, nutritional therapy, and phytotherapy. Many other medical systems may also be applied in IO, and the methods of these patient-centered holistic approaches overlap with one another. Many single treatment options of these CAM systems are safe, some have a good evidence base and are thus also adequate for integrative oncological care.

An IM treatment method with relatively good evidence supporting its efficacy is MBM that encompasses a variety of therapeutic techniques (e.g. psychological education, nutritional counseling, physiological exercise, and elicitation of the relaxation response and/or mindfulness) to enhance the patient’s natural capacity for mental and physical self-healing. MBM uses a holistic approach to health and healing that is supported by research on stress physiology/psychology, on psychoneuro-(endocrino-)immunology, and on the social and spiritual aspects of health. Several reviews of the use of MBM underline its efficacy in improving quality of life, immune function, the quality of sleep, and some psychological parameters in cancer patients [27-30]. Even a short-term MBM intervention of eight weeks can induce significant epigenetic changes, which may relate to long-term physiological effects [31]. Important contributions to MBM were made during the 1970s and 80s by prominent clinicians like Benson at Harvard Medical School [32,33] and Jon Kabat-Zinn at the University of Massachusetts Medical Center [34,35] and their coworkers. Benson focused on cognitive restructuring, relaxation techniques, exercise, diet, and social support, while Kabat-Zinn’s program emphasizes the role of meditation and the development of mindfulness in daily life. Breast cancer patients who participated in a mindfulness-based stress reduction (MBSR) program experienced a decrease in stress, depression, and other symptoms and were better able to cope with their illnesses [36].

### An example of IO in Germany - the practice at Kliniken Essen-Mitte

#### Set-up and clinical services

Since the beginning of 2010, the Breast Center (under the direction of PD Dr. Kümmel) and the Department of Internal and Integrative Medicine, Kliniken Essen-Mitte, academic teaching hospital of the University of Duisburg-Essen (Prof. Dr. Dobos), have been cooperating in patient care at the Kliniken Essen-Mitte. Each of the breast cancer patients treated there receives an individualized treatment plan based on the current literature and guidelines. The IO team, consisting of physicians and CAM therapists, reviews current international literature, guidelines, and health technology assessments relevant to every single breast cancer patient. Individual IO treatment plans are formulated based on detailed analyses of a patient’s case and the results of their individual tumor conferences, all contained in a database, as well as on SenoExpert.

**Figure 2:**
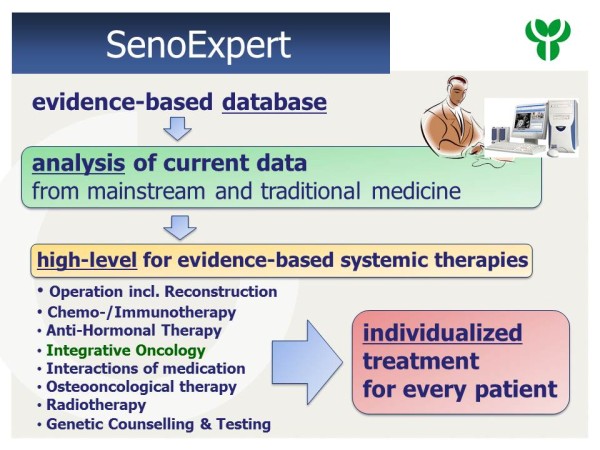
The concept of SenoExpert as an evidence-based database.

SenoExpert was introduced as a special database for breast cancer patients to aid in meeting the current scientific standards for high-quality patient care (see Figure 2). The database is continually up-dated by scientists, physicians, and MBM instructors who regularly review the medical literature and screen it for new guidelines. In addition, online conferences are held at regular intervals in which experts discuss anonymized breast cancer cases. The aim of these undertakings is to make the current guidelines and scientific evidence readily available to physicians responsible for the routine care of the patients.

The average duration of hospitalization is 3–4 days. Subsequently either the patient is treated in the out-patient clinic of the breast center, or the referring physician is sent detailed recommendations for the patient’s follow-up treatment.

**Figure 3:**
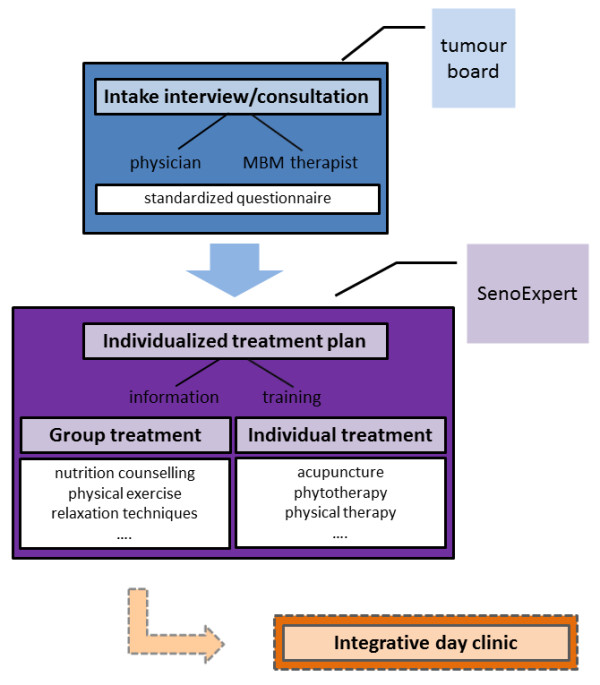
Components of Integrative Oncology at the Breast Center at the Kliniken Essen-Mitte, Germany.

In addition to receiving conventional treatment (see Figure 3), every patient in our department is routinely offered a consultation with a complementary and integrative medicine (CIM) physician and a MBM instructor. The CIM members of the IO team are trained in naturopathy, nutrition, sports pedagogic, and psycho-oncology, and they educate, and support patients in coping with their diseases and treatments. Each MBM consultation is based on the patient’s answers to a standardized questionnaire that inquires about the patient’s knowledge of her own diagnosis and its therapy, her previous experience with CIM and/or CAM methods, and her quality of life, psychological factors, and lifestyle. The questionnaire combines validated tools (Hamilton anxiety and depression scale, HADS; the EORTC QLC-C30 and EORTC QLC-BR23 of the European Organization for Research and Treatment of Cancer; the Brief Fatigue Inventory, BFI; and others) with items developed by one of our focus groups.

In 2010 and 2011, a total of 726 patients with the primary diagnosis of breast cancer were treated at the Department of Senology of the University of Duisburg-Essen. Beginning in October 2010, around 200 patients were additionally seen by CIM physicians who became part of the breast cancer team. The percentage of patients treated with CIM is steadily growing. Most of these patients presented with side effects from chemotherapy and/or endocrine treatment or the initial phases of anxiety and depression. The most frequent side effects reported were: gastrointestinal disorders, polyneuropathy, xerostomia, a fatigue syndrome during chemotherapy, joint pain and hot flashes (secondary to aromatase inhibitors or tamoxifen), depression and pain, and post-surgical hematoma or venous congestion. The CIM treatment for painful hematoma and venous congestion is leech therapy. According to our initial analysis, (ear, body) acupuncture reduced the side effects of endocrine therapy in 70% of our patients. Phytotherapy also helps to reduce the side effects of standard cancer treatments. Neuraltherapy, guasha massage, and cupping massage are employed to alleviate symptoms such as pain in scars or general post-surgical pain. In addition, physical exercisies, yoga, and training in mindfulness, relaxation, and nutrition are utilized to enhance the patient’s capacity for mental and physical self-healing. Mistletoe (*Viscum album*) and other treatments that have been shown to be efficacious, safe, and effective may also be applied. Thus, these integrative treatments are mostly applied to alleviate side effects, treat acute symptoms, and support patients in coping with their illness and in altering their lifestyle.

Additionally, the Department of Complementary and Integrative Medicine offers a day clinic program for oncology patients that is based on the principles of MBM. So far about 1500 patients have been treated within this setting. Our especially trained MBM instructors teach classical mindfulness concepts and offer modified mindfulness-based therapy classes (11 weeks, 6 hrs per meeting) that integrate nutritional seminars (lectures in a teaching kitchen), exercise training (walking, yoga, or qigong), and group support and self-care strategies (e.g. cupping massage, hydrotherapy). In addition, physicians specialized in naturopathy inform the patients about neuropathic treatments, and the nursing department provides information about methods such as wraps and cupping. The day clinic is mainly focused on consolidating techniques the patients have learned, enhancing mindfulness in daily-life, promoting self-care and self-knowledge, coping with the individual illnesses, and advising patients about lifestyle.

At present, several trials are being carried out to evaluate the ambulatory medical service at the Breast Center and the multimodal day clinic at our department. In addition, 6 and 12 months after completing their therapy, patients who have been treated at our clinic and consent to follow up are sent standardized questionnaires dealing with their QoL, psychological symptoms, dietary habits, and activities.

## Discussion

With CIM becoming very popular among cancer patients worldwide [14,17,37,38], we have established a breast cancer center according to the concept of IO in Germany.

One of the leading international centers in integrative oncology is the Memorial Sloan-Kettering Cancer Center in New York with its Integrative Medicine Service being the role model of our center [37]. This service is a horizontal academic unit that interacts with all other departments within the institution. A wide range of evidence-based complementary therapies including acupuncture, massage, music therapy, meditation, nutrition, counselling, and physical exercise are applied to optimize the patient’s overall treatment management [37].

The University of Texas M D Anderson Cancer Center’s Integrative Medicine Clinic is another world-leading cancer center with a long tradition in comprehensive cancer care [38]. Teams of experts from different disciplines collaborate on finding the best treatment plans for patients with various cancer diagnoses. An evaluation report revealed that most of their patients had advanced disease. In the authors’ view, CIM therapies came into play when the primary treatments had failed and the diseases had progressed, at which point the patients’ desire to try alternatives in treatment became more pronounced [38].

The concept of CIM is also successfully practiced in the Middle East. In 2008, an Integrative oncology program within the Clalit Health Organization’s oncology service at the Lin Medical Center, Haifa, Israel was established [39]. Its focus lies on supportive care during chemotherapy and advanced cancer using diet and supplements, herbal medicine, mind-body and touch therapies, acupuncture, AM, homeopathy and spiritual care [39].

In general, traditional medicine systems are very patient-oriented. By considering the traditional medicine backgrounds of patients, practitioners can engage in a deeper and more beneficial dialogue with their patients [14,39,40]. The need for a sensitive cross-cultural approach under consideration of social, cultural, and spiritual elements is particularly important in countries where patients’ beliefs derived from their cultural background and explanatory models of conventional medicine are difficult to reconcile [14]. Knowledge of the cross-cultural perspectives and co-existing health belief models of other cultures is of great value in treating the patients of these cultures.

In the Middle East, a collaboration of researchers of various countries met via the Middle East Cancer Consortium (MECC) to design an integrative oncology program based on a three-stage process [39,40]: After an initial historical and ethno-botanical search in Middle Eastern traditional medicine resources, a Medline search for CAM studies in cancer care in the Middle East was conducted, and finally, potentially useful herbs were selected for additional clinical research for this region. The focus lied in exploration of the potential role of Middle Eastern plants in cancer care by comparing these historical resources with contemporary scientific literature [41].

Unlike the in United States, funding for CAM research and its clinical application in Germany is very limited. While acceptance of CAM is generally growing and the decision makers of medical schools in German-speaking countries are positively inclined to integrate CAM into school curricula, only a small percentage of clinics and institutes have integrated CAM into their practices to date [17]. Compared with the international centers, our center in Essen is relatively small. However, unlike in other institutions, CIM therapies are directly implemented as a part of routine care in our breast center. CIM therapists and conventional caregivers work hand-in-hand.

Future challenges include extending the range of CIM therapies offered in our breast center and establishing further CIM practice in other oncological departments. All in all, the IO methods we practice are restricted to scientifically evaluated interventions. The majority of our patients appreciate the concept of IO, the treatments of which are partly covered by German insurance companies.

## Conclusion

Our IO model is well accepted by patients, and we have observed substantial benefits to them as a result of our treatments. However, further scientific evaluation of the specific add-on efficacy and cost-effectiveness of CAM treatments is urgently needed. Further research is also required to establish IO as an integral component of cancer treatment.

In our opinion, integrative care is crucial to the field of oncology and to establishing state-of-the-art breast cancer care. The potential of IM and IO lies in preventing illness and enhancing self-healing abilities, as well as in supporting mainstream treatment in order to speed up recovery processes, minimize side effects, and thereby reduce health-care costs.

## Summary

After many years of clinical and experimental practice in IM, we have achieved a great deal and have received recognition from patients and medical professionals. However, CAM treatments still need to be evaluated by more rigorous scientific methods. Three major factors must be considered in choosing a best treatment option: 1) the level of evidence supporting the efficacy of a treatment, 2) the cost of therapy, and 3) its safety, i.e., the existence of potential side effects. Western physicians and therapists are educated to question therapeutic strategies that are not based on rigorous evidence.

In our opinion the main challenge for IO at present is to analyze the complexities of the dynamic, multimodal, and individualized treatment strategies in integrative cancer care. Instead of concentrating solely on various trials of single treatment options, holistic strategies should be evaluated. However, scientific methods designed with settings appropriate for pharmaceutical trials are of limited scope for many IO treatment modalities. Randomized controlled trials, the scientific gold-standard, may be insufficient to address relevant questions about the outcomes of whole system strategies, since CAM therapies do not focus on isolated pathological processes, but rather on the whole patient with all of his or her complexities. CAM therapy systems are often based on long traditions and experience in patient care, while rigorous scientific evidence is often lacking. Substantial efforts are made to emerge clinical and laboratory investigations also for complementary therapies. In developing conventional drug therapies, laboratory investigations are carried out first, then phase I-III trials are conducted and finally clinical experience is gained. The order of clinical experience and systematic evaluation is reversed for CAM therapies on the one hand and conventional drug therapies on the other [42].

In most RCTs considerable efforts are made to avoid systematic bias. These efforts can cause a gap between the conditions of the traditional RCTs for the proof of efficacy and real-world healthcare. Well-designed observational studies in real-world conditions might enhance our understanding of the processes and provide better understanding of patient preferences and the underlying mechanisms in action. Although observational studies do not allow us to make conclusions about the cause of the efficacy of an intervention, they do help us gain knowledge about particular types of treatments and how they can be combined in standard care. We can observe whether patients get better overall and whether side effects occur. Observational studies and especially those that have a control group could provide useful data in the field of comparative effectiveness research. Systematic CIM assessments should utilize a broad array of diverse high-quality research methods and include randomized and non-randomized studies, cross-sectional studies, pragmatic trials, comparative effectiveness research, qualitative studies, case series, and socioeconomic analyses. But what we do need first and foremost is more systematic data on the safety and adverse side effects of CAM options. On a practical perspective, the experience shows that patients may not only use CAM option on the evidence-base. However, more such information will help physicians broaden their radius of operation and meet patients’ requests at once.

At the same time, we should consider that other criteria might apply for chronically ill patients. Many of the growing number of cancer survivors may profit from CAM options with low toxicity as an adjunct to their conventional treatments. Unlike researchers, patients are often not primarily interested in general physiological outcomes; rather, they want to regain control over their lives and enhance the quality of their lives. We hope that further trials investigating CAM modalities will underline the benefits we see in daily practice.

Future challenges lie in promoting the concept of IO in medical school curricula and in continuing education courses for oncologists, therapists, nurses, and family physicians. Only then will referring physicians, therapists, and nurses routinely recognize the patient’s need for holistic support.

## Abbreviations

AGO: Arbeitsgemeinschaft Gynäkologische Onkologie e.v.; AM: Anthroposophic medicine; CAM: Complementary and alternative medicine; CAM-Cancer: Concerted Action for Complementary and Alternative Medicine Assessment in the Cancer Field; CIM: Complementary and integrative medicine; IM: Integrative medicine; IO: Integrative oncology; MBM: Mind-body medicine; MBSR: Mindfulness-based stress reduction; NCCAM: National center for complementary and alternative medicine; NHIS: National health interview survey; NIH: National institutes of health.

## Competing interests

Part of the SenoExpert is financed by Roche, Sanofi Aventis, Cephalon and GSK. To help improve therapies, they provide research results on international communication platforms and forums. The companies have no influence on therapy recommendations.

All authors have disclosed any other financial or other relationships with any organization or entity with a financial interest in or in financial competition with the subject matter or materials discussed in the manuscript. Ms. Mary Passarge linguistically revised this manuscript.

## Authors’ contributions

GD, PV and KEC drafted the manuscript. All authors critically revised the paper for its content and read and approved the final manuscript.

## Pre-publication history

The pre-publication history for this paper can be accessed here:

http://www.biomedcentral.com/1471-2407/12/539/prepub
